# Chloride Permeability of Alkali-Activated Slag Concretes after Exposure to High Temperatures

**DOI:** 10.3390/ma17051028

**Published:** 2024-02-23

**Authors:** Baomeng Zhou, Qianmin Ma, Rongxin Guo, Ping Li

**Affiliations:** 1 Faculty of Civil Engineering and Mechanics, Kunming University of Science and Technology, Kunming 650500, China; 2 Yunnan Key Laboratory of Disaster Reduction in Civil Engineering, Kunming 650500, China; 3 International Joint Laboratory for Green Construction and Intelligent Maintenance of Yunnan Province, Kunming 650500, China

**Keywords:** alkali-activated slag concrete, high temperature, chloride permeability, pore structure

## Abstract

The number of fires in buildings and on bridges has increased worldwide in recent years. As a structural material, the strength of alkali-activated slag (AAS) concrete after exposure to high temperatures has been given much attention. However, research of its durability is still lacking, which limits the application of this type of concrete on a larger scale. In this context, as one of the most important aspects of durability, the chloride permeability of AAS concretes after exposure to high temperatures was examined in this study. The influence of the alkali concentration (Na_2_O%) and the modulus (Ms) of the activator, as well as the influence of heating regimes, including the heating rate, duration of exposure to the target temperature, and cooling method, was also discussed. The results show that the chloride permeability of the AAS concretes increased with temperature elevation. Due to the interference of pore solution conductivity, the influence of the Na_2_O% and the Ms of the activator on the chloride permeability of the AAS concretes was not made clear by using the ASTM C 1202 charge passed method; however, after exposure to high temperatures, AAS with a lower Na_2_O% and lower Ms has lower porosity and may have lower chloride permeability, which needs further investigation. Faster heating for a longer duration at the target temperature and water cooling reduced the resistance of the AAS concretes to chloride permeability as a result of their increased porosity.

## 1. Introduction

Alkali-activated slag (AAS) is a type of cement-free cementitious material where ground granulated blast furnace slag (GGBS) is activated using an alkali activator (such as sodium silicate solution, namely, water glass: WG) [1,2]. Due to its effective reduction in the consumption of non-renewable resources and the emission of greenhouse gases, as well as its high strength and excellent durability, there is significant potential to use AAS as an environmentally friendly cementitious material in the manufacture of structural concrete in construction.

The number of fires in buildings and bridges has increased worldwide in recent years. The temperature of a building during a fire can reach hundreds or even thousands of degrees; the structural safety and service life of the building is reduced as a result of physio-chemical phenomena such as structural expansion, shrinkage, thermal cracks, and hydrate decomposition that occur during exposure to high temperatures. This means that the design life of the general building structure, usually 50 years, cannot be reached. If a construction is to continue to be in use after a fire, especially concrete structures in chloride environments, such as residential buildings in coastal areas, and sea-crossing bridges, its residual strength and durability should be fully understood. In terms of the residual strength of concrete after exposure to high temperatures, it was found that with the elevation of temperature, the strength of Portland cement (PC) concrete decreases [3], as does AAS concrete [4,5,6,7,8,9,10,11]. Another aspect is chloride permeability as an important evaluation of durability, and studies of this also concluded that the chloride permeability of PC concrete increases generally with the elevation of temperature as a result of the deterioration of the microstructure [12,13,14,15,16]. With respect to AAS, Gutierrez et al. used the ASTM C 1202 test to evaluate the charge passed of AAS mortar with an increase in temperature from room temperature to 1000 °C [17]. It was found that even up to 700 °C, the charge passed of the AAS mortar increased no more than four times compared with that at room temperature (from approximately 1600 C to 5500 C), and the values were always lower than that of PC following exposure to elevated temperature. Above 1000 °C, the charge passed of the AAS mortar increased dramatically, up to approximately 21,000 C, which was nearly twice as high as that of the PC mortar. Fu et al. used the RCM method to test the chloride penetration coefficient of AAS mortar with the elevation of temperature [18]. It was found that after 200 °C, the coefficient of the AAS mortar was not only lower than that of PC mortar but also lower than its value at room temperature. However, as a result of an increase in porosity, especially the fraction of the pores with sizes of 0.1 μm to 1 μm, the coefficient value of the AAS mortar dramatically increased afterward. After 600 °C, the value was one order of magnitude higher than the value at room temperature.

From this brief review, it can be seen that the chloride permeability of AAS after exposure to high temperatures has scarcely been studied. The existing studies have only focused on mortar and not concrete specimens, which has limited the guidance on structural construction. Furthermore, as one of the crucial factors influencing the chloride permeability of AAS concrete at room temperature [19], the effect of alkali concentration (Na_2_O%) and the modulus (Ms) of WG on the chloride permeability of AAS concrete after exposure to high temperatures should also be clearly identified. 

On the other hand, heating regimes, such as the heating rate, duration of exposure at the target temperature, and cooling method, have a considerable influence on the strength of AAS after exposure to high temperatures. Ma et al. [20] found that extending the duration of AAS concrete at a target temperature decreased its strength, and after 400 °C, the increase in the heating rate also increased the loss of strength. The results of Akçaözoğlulu et al. show that water cooling exacerbated the temperature difference between the inside and outside of the specimen, and serious deterioration of the mechanical properties of AAS mortars was observed [21]. Ma et al. [20] showed that water cooling caused a larger loss in the strength of AAS concrete compared to natural cooling. However, the influence of heating regimes on the chloride permeability of AAS after exposure to high temperatures has been scarcely reported.

Therefore, considering the lack of research in this respect, the current study examines the effect of alkali concentration and modulus of WG, as well as different heating regimes, on the chloride permeability of AAS concrete, which can provide a research theory for the high-temperature resistance of this type of concrete and can also provide a basis for the repair and reinforcement of buildings after fire, widening the scope of AAS concrete applications in structural engineering.

## 2. Experimental Program

In this study, AAS concretes with alkali concentrations (Na_2_O% mass of slag) of 4, 6, and 8 and moduli (SiO_2_/Na_2_O molar ratio) of WG of 1.0, 1.5, and 2.0 were manufactured to study the influence of high temperatures on their chloride permeability. Furthermore, the influence of heating regimes, including heating rate (5 °C/min, 10 °C/min), duration of exposure at a targeted temperature (2 h, 1 h), and cooling method (water cooling, natural cooling), on the chloride permeability of the concretes was also studied. In order to better understand the influence of high temperature on the chloride permeability of the AAS concretes, the evolution of the pore structure of AAS pastes with temperature elevation was also studied. PC counterparts with the same binder content, water/binder ratio (W/B), and sand ratio were manufactured as a reference.

### 2.1. Materials

GGBS from Qujing, Yunnan, China, with a grade of S75 was used to manufacture the AAS concretes and paste specimens. Chemical composition was determined using an X-ray fluorescence spectrometer; its chemical composition is given in Table 1. Ordinary Portland cement with a strength grade of 42.5, which was manufactured by Huaxin Cement Co., Ltd. in Kunming, China, was used to manufacture the PC counterparts. Its chemical composition is also given in Table 1. From Table 1, it can be seen that the chemical composition of GGBS and PC is mainly CaO, SiO_2_, and Al_2_O_3_, accounting for more than 80% of the total chemical composition, but the CaO content of PC is much higher than that of GGBS.

WG from Kunmin, Yunnan, China, with Na_2_O% and SiO_2_% of 7.51% and 22.22%, respectively, was used as an alkali activator. NaOH produced by Fuchen Chemical Reagent Co., Ltd. (Tianjin, China), with a purity of not less than 96%, was added to the WG to change the content of Na_2_O% and the Ms of the WG to the required values.

Limestone with a continuous gradation of 5–25 mm was used as a coarse aggregate (CA). Manufactured sand with a fineness modulus of 2.82 was used as a fine aggregate (FA) that was sieved before mixing to remove stone powder. 

Tap water was used for mixing. A polycarboxylic polymer-based superplasticizer (from Fuchen, Tianjin, China) was used to mix the PC mixtures, whose dosage was selected as 0.08% after a trial. 

### 2.2. Mix Proportions

AAS concretes with Na_2_O% of 4, 6, and 8 and Ms of water glass of 1.0, 1.5, and 2.0 were manufactured. The binder content, which was the sum of the GGBS content and the solid content of the adjusted WG, was kept constant at 380 kg/m^3^ for all mixes. A W/B of 0.45 was used for all of the mixes, and the water content in the WG was taken into account. A sand ratio of 40% was used. The mix proportions of the concretes are given in Table 2. For the purpose of comparison, PC concrete was also prepared as a reference, whose binder content, W/B, and sand ratio were kept the same as those used for the AAS concretes. Paste specimens were also prepared for pore structure analysis, where a W/B of 0.3 was used.

### 2.3. Specimen Preparation

**Figure 1:**
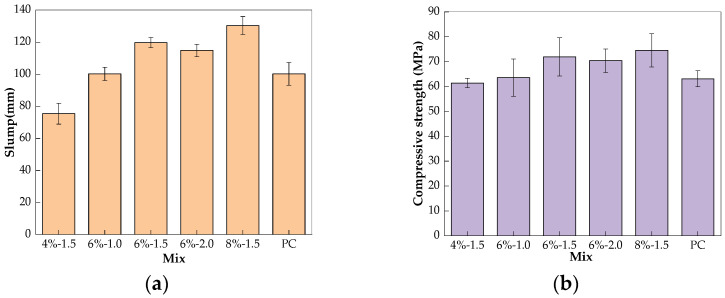
Slump (**a**) and compressive strength (**b**).

Concrete mixing was conducted following the “Standard for test method of performance on ordinary fresh concrete” GB/T 50080-2016 [22]. One day before mixing, half of the additional water was used to dissolve NaOH; the solution was then mixed with WG to adjust its modulus. In order to ensure the quality of the mix proportion, all mixes achieved similar compressive strength grades at 90 days, of 60–70 MPa, and the slump was measured; the results are provided in Table 3 and Figure 1.

The concrete mixture was cast into molds in two layers. After each layer casting, the mixture, with the mold, was placed on a vibration table to be vibrated until no bubbles were generated at the surface of the mixture. After demolding 24 h later, the specimens were placed in a standard curing room with a temperature of 20 ± 2 °C and a relative humidity of 95% for 90 days. The sizes of the specimens were Ф 100 mm × 200 mm and 100 mm × 100 mm × 100 mm for the chloride permeability and compressive strength tests, respectively, and three parallel specimens were prepared for each test at each temperature. After curing, the tops and bottoms of the specimens for the chloride test were cut off and the middle parts of the specimens, with sizes of Ф 100 mm × 50 mm, were used for the test. For the purpose of quality control, concrete specimens with sizes of 100 mm × 100 mm × 100 mm were prepared as well to test their compressive strength and slump at room temperature; the results are provided in Table 3 and Figure 1. The paste specimens were mixed in accordance with “Test methods for water requirement of normal consistency, setting time and soundness of the Portland cement” GB/T 1346-2011 [23]. After mixing, the mixture was cast into a mold measuring 25 mm × 25 mm × 25 mm, followed by vibration to make the mixture dense. The same curing regime used for the concrete was applied to the paste.

### 2.4. Heating Regime

**Figure 2:**
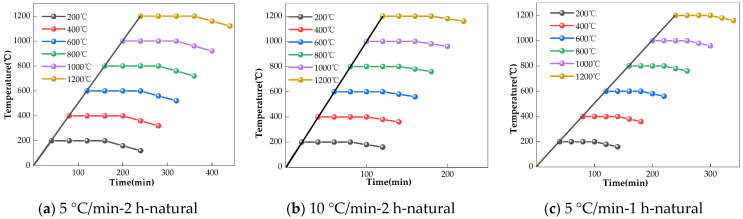
Heating regime.

The concrete and paste specimens were heated in a muffle furnace to temperatures of 200 °C, 400 °C, 600 °C, 800 °C, and 1200 °C, respectively. The heating regime benchmark was a heating rate of 5 °C/min. When the target temperature was achieved, the temperature was maintained for 2 h; then, the power was tuned off and the specimens were cooled in the furnace naturally to room temperature. In order to study the influence of the heating regime on the chloride permeability of the concretes after exposure to high temperatures, an additional regime involving a heating rate of 10 °C/min, a duration at the target temperature of 1 h, and water cooling was implemented on the AAS mix 6%-1.5 for comparison; see Figure 2. Water cooling refers to the process of taking out the specimens immediately after heating and then placing them in a sink of water until room temperature was reached.

### 2.5. Tests

In accordance with the “Standard for test methods of long-term performance and durability of ordinary concrete” GB/T 50082-2009 [24], the ASTM C 1202 method was used to evaluate the resistance of the concretes to chloride at room temperature and after temperature elevation. Before testing, the specimens were water-saturated in a vacuum. Paraffin was applied onto the surfaces of the samples except the two test surfaces. One of the test surfaces faced an upstream cell (cathode) containing 0.55 M NaCl solution, and the other surface faced a downstream cell (anode) containing 0.30 M NaOH solution. Copper electrodes were inserted into both cells. A voltage of 60 V was applied across the specimen by connecting the anode and the cathode electrodes to the negative and positive terminal leads of the power supply, respectively. The current flow was monitored during the test. The integral of the current during the first six hours of the test, namely, charge passed, was used to evaluate the chloride permeability of the specimen. A higher charge passed value usually suggests a higher chloride permeability. The result was reported as an average of three specimens.

Unfortunately, the specimens cracked significantly after 600 °C exposure, resulting in the charge passed value exceeding the upper limitation of the instrument. Therefore, the ASTM C 1202 test was not carried out on the specimens exposed to conditions of 600 °C and higher. Mercury intrusion porosimetry (MIP) was used to characterize the pore structure of the paste specimens at room temperature and after temperature elevation, where an Micromeritics Auto Pore IV 9500 model (from Micromeritics, Norcross, GA, American) was applied. The principle of the test is to press mercury into the pores of the specimen and then obtain the parameters of the pore structure by calculating the amount of mercury injected and the pressure used. The paste specimens were crushed, and a fragment from the inner part with a size of approximately 5 mm was taken and its hydration terminated by soaking in absolute ethanol for 3 days. Following this, the fragment was dried in a vacuum drying dish until the test. The PC specimen was severely damaged after 1200 °C, and as it was so loose, the MIP test could not be performed.

## 3. Results and Discussion

### 3.1. Influence of Na_2_O% and Ms

**Figure 3:**
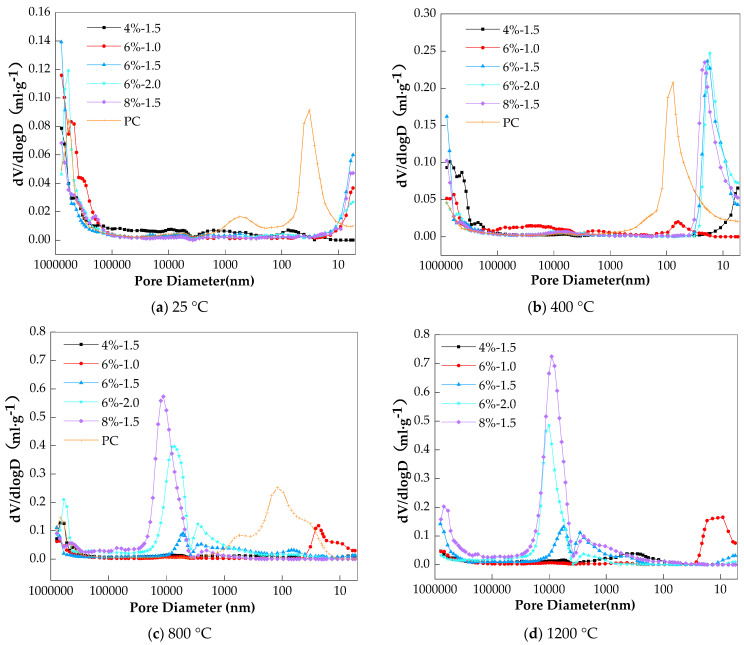
Pore size distribution of AAS pastes exposed to different elevated temperatures.

The charge passed of the concrete specimens at room temperature and those after temperature elevation are given in Table 4. From the table, it can be seen that the charge passed of both the AAS and PC concretes significantly increase with the increase in the exposure temperature, indicating that the chloride permeability of these concretes increased due to heating. The porosity and pore size distribution of the corresponding paste specimens were obtained from the MIP test, and the results are expressed in Table 5 and Figure 3, respectively. From Table 5, it can be seen that with the temperature elevation, the total porosity of all mix proportions of concrete increases, which could be the reason for the increased chloride permeability. This conclusion was also put forward in the study by Fu et al. [18]. Further, from Figure 3, it can be seen that for any given specimen type, the peaks of the curves generally shift leftward with temperature, indicating that the pores have been coarsened with the increase in temperature. Through the analysis of SEM images of AAS paste after exposure to high temperatures, Ma et al. [20] also found that the pores were coarsened with the increase in the temperature, and this could also be responsible for increased chloride permeability.

Compared to the PC samples, fewer pores were detected in the AAS, as shown in Table 5, and the pores in the AAS were finer at room temperature and even after 400 °C (the peaks of the AAS are on the right side of those of the PC, see Figure 3). As a result, the resistance of AAS concretes to chloride permeability should be much better than that of PC concrete both at room temperature and exposure above 400 °C. Fu et al.’s study also proposed this conclusion [18]. However, from Table 4, it can be seen that the charge passed of all AAS mixes is higher than that of PC after 400 °C. Even with room temperature storage, which is the most favorable for giving a low-permeability pore structure, the charge passed of the AAS mixes was comparable with that of PC, with some AAS mixes even exhibiting a significantly higher charge passed than that of PC. It is known that charge passed is a reflection of both the pore structure and pore solution conductivity of specimens [9]. A high charge passed value could be a result of a poorly developed pore structure and/or the high conductivity of the pore solution. It has been confirmed that AAS has higher pore solution conductivity than that of PC [19]. Therefore, the higher charge passed of the AAS concretes observed here could be caused by their higher pore solution conductivity rather than higher chloride permeability.

**Figure 4:**
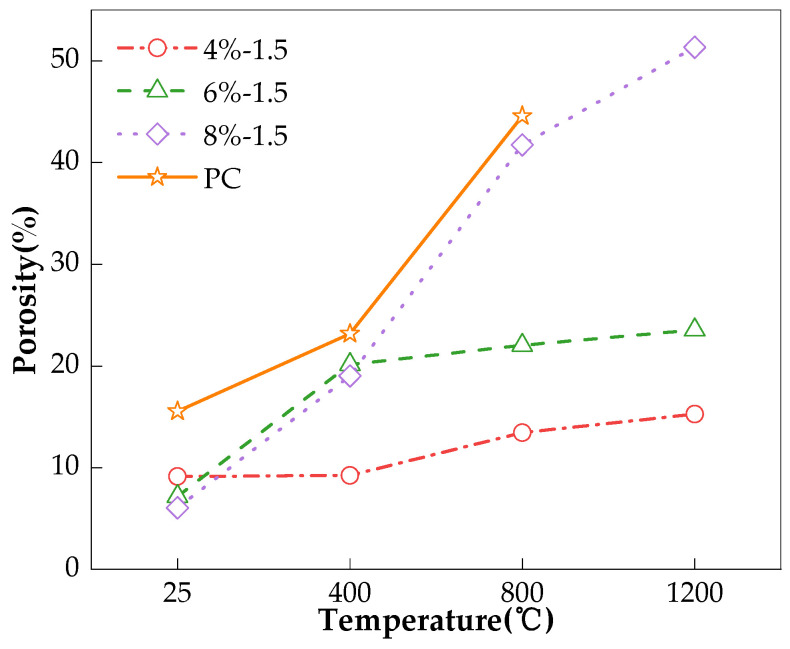
Effect of Na_2_O% on porosity of pastes.

**Figure 5:**
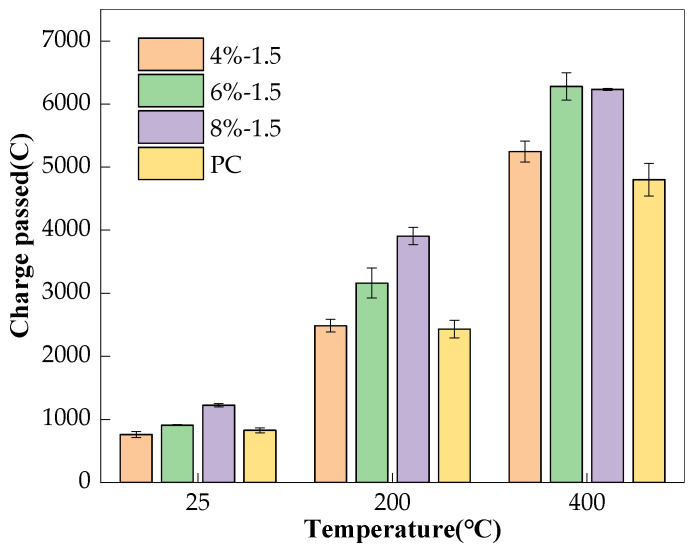
Effect of Na_2_O% on charge passed of concretes.

Similarly, as shown in Figure 4, with the increase in Na_2_O%, the AAS concretes at room temperature had a lower porosity as a result of sufficient reaction [25,26,27,28,29,30], therefore, indicates that these samples should have lower chloride permeability. However, the AAS concretes with more Na_2_O% yielded higher charge passed, indicating higher chloride permeability, as shown in Figure 5. In this context, it could be deduced that the pore solution conductivity dominated the charge passed rather than the pore structure, since the pore solution conductivity of AAS increased significantly with Na_2_O%. After exposure to high temperatures, the pore structure of AAS became significantly damaged, as illustrated in Figure 4. Further, probably due to the AAS with higher Na_2_O% being prone to shrinkage [31], the AAS concretes being exposed to high temperatures could have intensified the shrinkage. Therefore, different from room temperature, the porosity of the AAS generally increased with the increase in Na_2_O% after exposure to high temperatures, which, therefore, dominated the charge passed of the AAS concrete, which seems to indicate that AAS concretes with the lowest Na_2_O have the best relative performance when exposed to high temperatures. However, the experimental results of Rashad et al. [32] showed that Na_2_O% had no obvious effect on the performance of AAS paste after high temperature.

**Figure 6:**
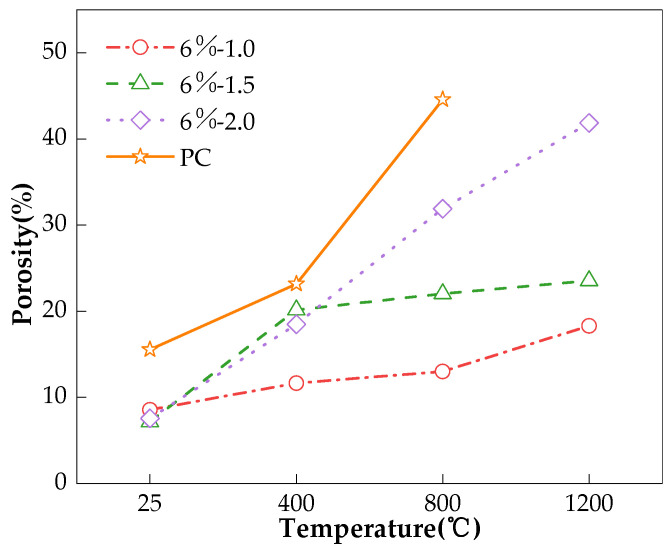
Effect of Ms on porosity of pastes.

**Figure 7:**
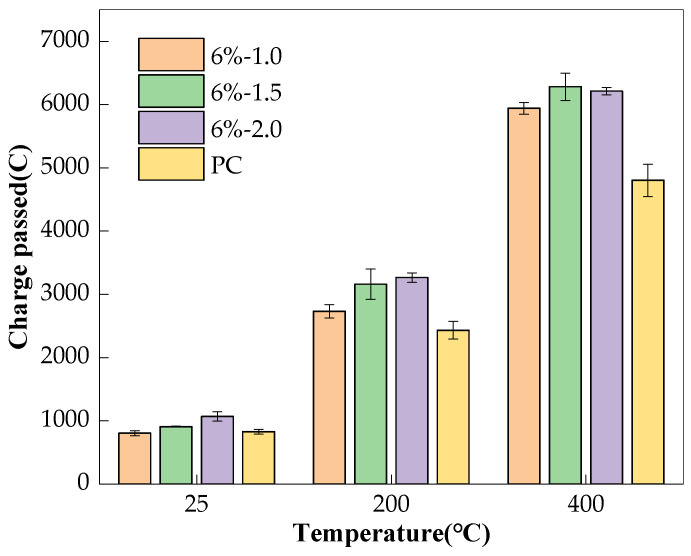
Effect of Ms on charge passed of concretes.

This is also true for the influence of Ms on the charge passed and porosity of the AAS mixes, as given in Figure 6 and Figure 7, respectively. At room temperature, higher Ms shortens the hydration induction period of the slag and can provide more SiO_4_^4−^ ions to accelerate the production of hydration products [27], and therefore, there is lower porosity; see Figure 6. The sample should have a lower chloride permeability. However, since the conductivity of the pore solution dominated the charge passed, the higher Ms yielded a higher charge passed, as shown in Figure 7. After exposure to high temperatures, the pore structure of the AAS concrete deteriorated; meanwhile, the higher Ms, fuller hydration reaction, and greater shrinkage of the AAS concretes led to the pore structure becoming significantly damaged, and there was a higher charge passed, as shown in Figure 6 and Figure 7. The experimental results of Guerrieri et al. [33] also showed that Ms had no obvious effect on the performance of the AAS paste after high temperature.

Overall, at room temperature and after high temperatures, due to the influence of the pore solution conductivity, the ASTM C 1202 method to test the chloride permeability of the AAS concretes remained unclear. However, this information may be obtained from the porosity results: AAS concrete has lower chloride permeability at lower Na_2_O% and Ms when exposed to high temperatures; therefore, the influences of the activator (Na_2_O% and Ms) need further investigation using a proper chloride test approach instead.

### 3.2. Influence of Heating Regime

**Figure 8:**
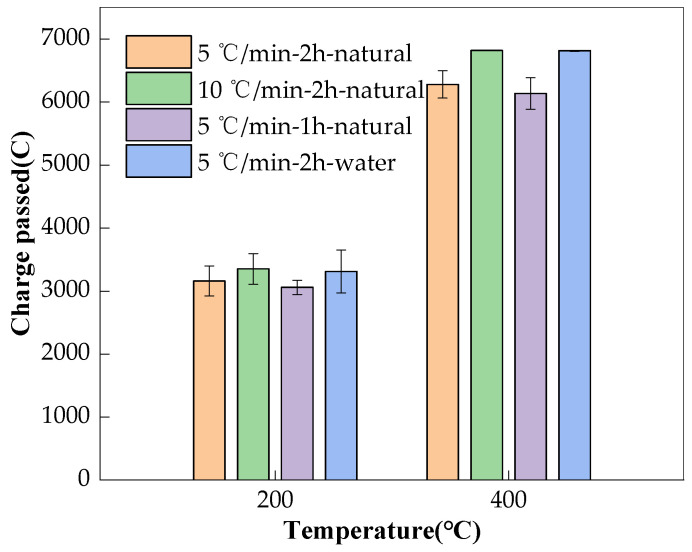
Influence of heating regime on charge passed of the AAS concretes.

**Figure 9:**
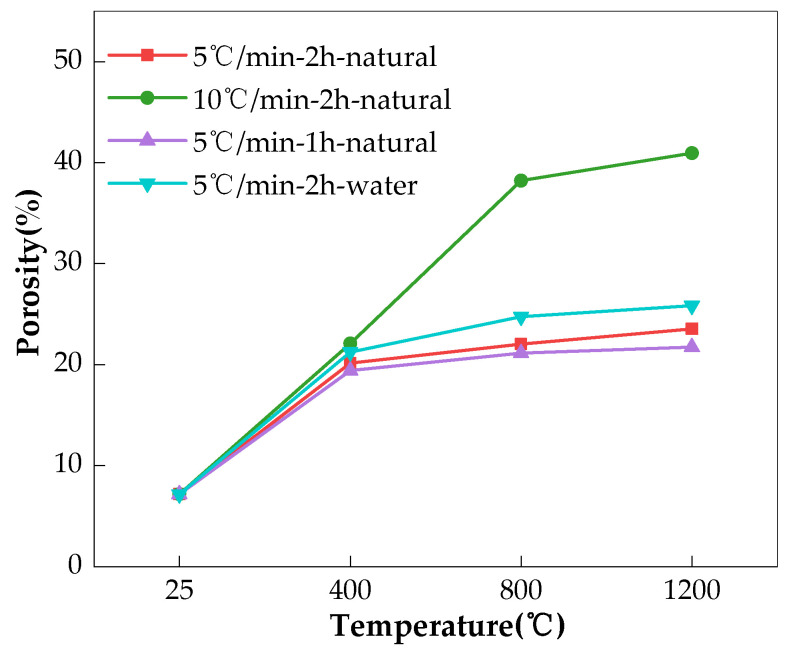
Influence of heating regime on porosity of the AAS pastes.

The influence of the heating rate (10 °C/min, 5 °C/min), duration of exposure at the target temperature (2 h, 1 h), and cooling method (water cooling, natural cooling) on the charge passed of the AAS concretes is illustrated in Figure 8. It can be seen that the concrete that was heated faster always gave a higher charge passed, indicating a lower resistance to chloride permeability. The reason for this could be that the faster heating enlarged the difference in temperature between the surface and the internal part of the specimen. Thermal stress then formed, resulting in the deterioration of the pore structure, as shown in Figure 9, and accelerating the charge passing through; these findings are consistent with those of Fu [18]. Further, shortening the duration of the specimens’ exposure to the target temperature reduced the charge passed, indicating reduced the chloride permeability. A shorter duration at the target temperature could reduce thermal damage to the structure of the specimen, leading to lower porosity [34], as shown in Figure 9. This could be the reason for the lower chloride permeability. Compared to natural cooling, water cooling sharply reduced the temperature of the surface of the specimens in a shorter time, which could have increased the temperature difference between the surface and the inner part of the specimens. Then, thermal stress could have been generated to induce cracking to allow more charge passing (see Figure 8 and Figure 9), as illustrated in Figure 8 [20,35,36].

Figure 9 further shows that the heating rate was the most responsible for the thermal deterioration of the pore structure, followed by the cooling method. The duration at the target temperature had the smallest influence. These findings were especially true when the temperature increased to 800 °C and above. Though the charge passed test was only conducted up to 400 °C, similar results still began to emerge, and it is not difficult to presume that if the test could be carried out after a higher temperature exposure, the findings would be much clearer.

## 4. Conclusions

This study focused on the chloride permeability of AAS concretes exposed to high temperatures, particularly with regard to the effect of the Na_2_O%, Ms, and heating regime on the chloride permeability. Based on the experimental program used in this study, the following findings could be concluded:

(1) The resistance of AAS concretes to chloride ingress was reduced with the increase in temperature as a result of both increased total porosity and pore coarsening.
(2) The porosity results imply that, after exposure to high temperatures, AAS concrete with a lower Na_2_O% and lower Ms may exhibit lower chloride permeability; however, the charge passed of the AAS concretes examined in this study could have been caused by pore solution conductivity rather than chloride permeability, and it is therefore not suitable to apply the ASTM C 1202 charge passed method to compare the chloride permeability of concretes with different cementitious systems, such as PC and AAS, or different AAS concrete mixes. These findings should be confirmed using a proper approach instead, such as the non-steady-state chloride migration test (NT BUILD 492).
(3) Faster heating and water cooling following the high-temperature exposure in this study could have increased the temperature gradient between the surface and the internal part of the AAS concrete, aggravating the coarsening of the pore structure and then reducing the resistance of the concrete to chloride ingress. Shortening the duration of the AAS concretes’ exposure to the target temperature may have reduced the thermal damage to the pore structure, thus yielding better resistance to chloride ingress.

## 5. Future Prospects

(1) Future studies may use a proper approach to confirm the results of this study, such as the non-steady-state chloride migration test (NT BUILD 492).
(2) After a large number of studies have been conducted, artificial neural network models could be utilized in the prediction and evaluation of the impermeability performance of AAS concretes after exposure to high temperatures.

## Figures and Tables

**Table 1:** Chemical compositions of GGBS and PC (oxide%).

	CaO	SiO_2_	Al_2_O_3_	MgO	TiO_2_	SO_3_	MnO	Na_2_O	K_2_O	Fe_2_O_3_
GGBS	38.26	31.40	15.71	6.90	2.94	1.43	1.23	0.96	0.53	0.38
PC	60.45	20.44	5.59	3.41	1.18	4.30	0.06	0.32	0.77	3.21

**Table 2:** Mix proportion of the concretes.

Mix No. (Na_2_O%-Ms)	kg/m^3^							
	GGBS	PC	WG	NaOH	CA	FA	Water	Superplasticizer
4%-1.5	344.2	-	90.0	9.1	1138.8	759.2	107.8	-
6%-1.0	336.4	-	87.9	17.5	1142.7	761.8	109.2	-
6%-1.5	328.7	-	128.9	13.0	1164.5	776.3	80.5	-
6%-2.0	321.5	-	168.0	8.6	1185.4	790.2	53.0	-
8%-1.5	314.6	-	164.4	1.5	1188.0	792.0	55.5	-
PC	-	380	-	-	1079.4	719.6	171.0	2.0 × 10^−3^

**Table 3:** Slump and 90 d compressive strength of concretes.

Mix No.	Slump (mm)	Compressive Strength (MPa)
4%-1.5	75.4 (±6.5)	61.4 (±1.9)
6%-1.0	100.2 (±4.1)	63.6 (±7.5)
6%-1.5	119.7 (±3.2)	71.9 (±7.7)
6%-2.0	114.8 (±3.9)	70.4 (±4.7)
8%-1.5	130.3 (±5.6)	74.5 (±6.7)
PC	100.2 (±7.1)	63.1 (±3.2)

**Table 4:** Charge passed of the concrete specimens.

Mix No.	Temperature	Charge Passed (C)
4%-1.5	25 °C	759 (±47.5)
	200 °C	2485 (±101.8)
	400 °C	5247 (±166.0)
6%-1.0	25 °C	804 (±39.0)
	200 °C	2731 (±105.8)
	400 °C	5940 (±94.9)
6%-1.5	25 °C	908 (±7.0)
	200 °C	3161 (±238.9)
	400 °C	6280 (±218.9)
6%-2.0	25 °C	1068 (±74.0)
	200 °C	3263 (±74.8)
	400 °C	6212 (±59.6)
8%-1.5	25 °C	1224 (±24.9)
	200 °C	3907 (±135.9)
	400 °C	6233 (±18.5)
PC	25 °C	827 (±38.6)
	200 °C	2431 (±140.4)
	400 °C	4801 (±258.2)

**Table 5:** Porosity of the paste specimens (%).

Mix No.	25 °C	400 °C	800 °C	1200 °C
4%-1.5	9.14	9.25	13.43	15.27
6%-1.0	8.57	11.66	13.01	18.31
6%-1.5	7.17	20.15	22.03	23.54
6%-2.0	7.56	18.49	31.90	41.86
8%-1.5	6.05	19.01	41.74	51.34
PC	15.54	23.18	44.58	-

## Data Availability

Data are contained within the article.

## Abbreviations

PC	Portland cement
AAS	Alkali-activated slag
GGBS	Ground granulated blast furnace slag
Na_2_O%	Alkali concentration of activator
Ms	Modulus of activator
CA	Coarse aggregate
FA	Fine aggregate
WG	Water glass
W/B	Water/binder ratio
C	Charge passed
